# Structural snapshots of *Mycobacterium tuberculosis* enolase reveal dual mode of 2PG binding and its implication in enzyme catalysis

**DOI:** 10.1107/S2052252523008485

**Published:** 2023-10-21

**Authors:** Mohammed Ahmad, Bhavya Jha, Sucharita Bose, Satish Tiwari, Abhisek Dwivedy, Deepshikha Kar, Ravikant Pal, Richard Mariadasse, Tanya Parish, Jeyaraman Jeyakanthan, Kutti R. Vinothkumar, Bichitra Kumar Biswal

**Affiliations:** a Structural and Functional Biology Laboratory, National Institute of Immunology, Aruna Asaf Ali Marg, New Delhi 110067, India; b Department of Zoology, GDM Mahavidyalaya, Patliputra University, Patna 800020, India; c Institute for Stem Cell Science and Regenerative Medicine, Bangalore 560065, India; d Department of Bioinformatics, Alagappa University, Karaikudi, Tamil Nadu 630003, India; e Infectious Disease Research Institute, 1616 Eastlake Avenue E, Suite 400, Seattle, WA 98102, USA; f Seattle Children’s Research Institute, Seattle, WA 98109, USA; g National Centre for Biological Sciences, Tata Institute for Fundamental Research, Bangalore 560065, India; Chinese Academy of Sciences, China

**Keywords:** *Mycobacterium tuberculosis*, enolase, gluconeogenesis, cryo-electron microscopy, X-ray crystallography, enzyme mechanisms

## Abstract

Enolase, a ubiquitous enzyme, catalyzes the reversible conversion of 2-phospho­glycerate (2PG) to phospho­enolpyruvate (PEP) in the glycolytic pathway of organisms of all three domains of life. The underlying mechanism of the 2PG to PEP conversion has been studied in great detail in previous work, however that of the reverse reaction remains to be explored. Here we present structural snapshots of *Mycobacterium tuberculosis* (*Mtb*) enolase in apo, PEP-bound and two 2PG-bound forms as it catalyzes the conversion of PEP to 2PG. The two 2PG-bound complex structures differed in the conformation of the bound product (2PG) *viz* the widely reported canonical conformation and a novel binding pose, which we refer to here as the alternate conformation. Notably, we observed two major differences compared with the forward reaction: the presence of Mg_B_ is non-obligatory for the reaction and 2PG assumes an alternate conformation that is likely to facilitate its dissociation from the active site. Molecular dynamics studies and binding free energy calculations further substantiate that the alternate conformation of 2PG causes distortions in both metal ion coordination and hydrogen-bonding interactions, resulting in an increased flexibility of the active-site loops and aiding product release. Taken together, this study presents a probable mechanism involved in PEP to 2PG catalysis that is likely to be mediated by the conformational change of 2PG at the active site.

## Abstract 2

Enolase catalyzes the reversible conversion of 2-phosphoglycerate to phosphoenolpyruvate. Structural snapshots of *Mycobacterium tuberculosis* enolase provide critical insights into the mechanism of conversion of phosphoenolpyruvate to 2-phosphoglycerate.

## 1. Introduction

*Mycobacterium tuberculosis* (*Mtb*), the causative agent of tuberculosis (TB) in humans, is an ever-evolving bacterium capable of surviving in adverse host microenvironments by evading a vast array of host immune responses. Although there is a clinical regime to treat TB, the emergence of multi-drug resistant (MDR) and extensively drug-resistant (XDR) strains of the bacterium and the recurrent co-infection with HIV have made TB a global health concern. In this context, novel drugs that can target the primary metabolic pathways of the bacterium and prove to be effective on the drug resistant strains are highly sought after.

Glycolysis is one of the highly conserved metabolic pathways among all life forms. Enolase catalyses the ninth step of glycolysis, *i.e.* the conversion of 2-phospho­glycerate (2PG) to phospho­enolpyruvate (PEP), and the reverse reaction in gluconeogenesis. Additionally, enolase is a well known moonlighting protein with other functions within the cytoplasm and on the cell surface (Henderson & Martin, 2011 ▸). It is reported to play a role in pathogenesis of *Streptococcus pyogenes* (Walker *et al.*, 2005 ▸), *Candida albicans* (Jong *et al.*, 2003 ▸; Silva *et al.*, 2014 ▸), *Mtb* (Rahi *et al.*, 2017 ▸), *Leishmania sp.*, *Trypanosoma cruzi*, *Trypanosoma. brucei* (Avilán *et al.*, 2011 ▸), *Aphidius ervi* (Falabella *et al.*, 2009 ▸), *Bacillus anthracis* (Agarwal *et al.*, 2008 ▸), *Streptococcus pneumoniae* (Bergmann *et al.*, 2001 ▸) *etc*. In *E. coli*, enolase is reported to be essential for RNA processing and decay (Kaberdin & Lin-Chao, 2009 ▸). The varied localizations and crucial functions of enolase make it a potential molecular drug target. Enolase is essential for the growth of *Mtb in vitro* (Sassetti *et al.*, 2003 ▸; Griffin *et al.*, 2011 ▸). 2-Amino­thia­zoles, which have been reported to exhibit bactericidal activity against *Mtb*, target enolase (Wescott *et al.*, 2018 ▸). Collectively, these studies prompted us to study *Mtb* enolase (MtEno) in order to develop novel anti-TB inhibitors. We thus pursued a structure-guided approach and attempted to derive a mechanistic understanding of the catalysis by MtEno. We grew diffraction-quality crystals of apo MtEno and its substrate-/product-bound complexes and elucidated their structures. Interestingly, to our knowledge, these structures provide the first insights into the underlying mechanism of the reverse reaction: the conversion of PEP to 2PG.

**Figure 1:**
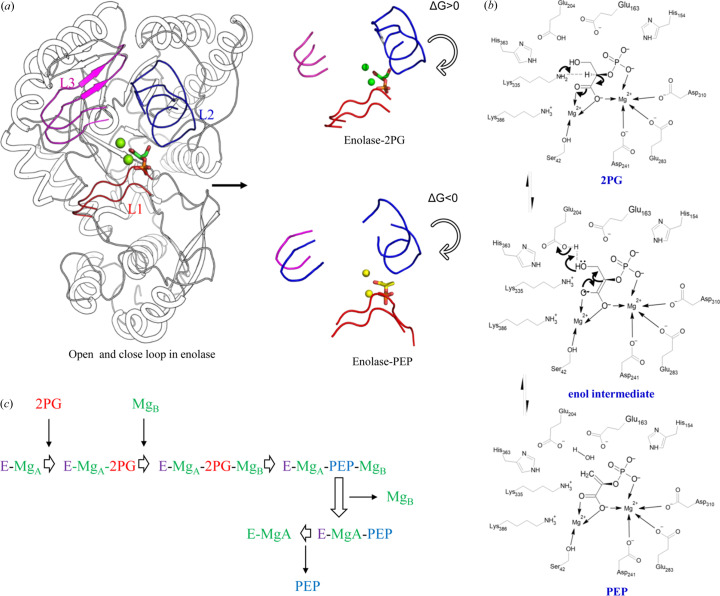
General mechanism of enolase in forward catalysis. (*a*) Opening and closing of the active site loops in enolase. The loop opening is thermodynamically more favorable in the PEP-bound than the 2PG-bound enzyme. Reprinted (adapted) with permission from Li & Hammes-Schiffer (2019 ▸). Copyright 2019 American Chemical Society. (*b*) General catalytic mechanism for forward catalysis. (*c*) Catalytic cycle for the forward reaction.

The forward catalysis has been described extensively using a number of homologs of MtEno (Lebioda & Stec, 1991 ▸; Reed *et al.*, 1996 ▸; Zhang *et al.*, 1997 ▸). The active site of enolase contains a conserved domain made of (1) three dynamic loops that close on substrate binding [Fig. 1 ▸(*a*)], (2) a firmly coordinated magnesium ion (Mg_A_) that interacts with the carboxyl­ate group of the ligand, (3) a transiently bound magnesium ion (Mg_B_) that coordinates primarily to the phosphate group of the ligand and the β-OH group of a serine residue, and (4) charged residues that are involved in proton abstraction and water removal from 2PG to form PEP [Fig. 1 ▸(*b*)]. The catalysis starts with the binding of Mg_A_ while the loops remain open. The substrate 2PG, in a widely reported conserved canonical binding pose (2PG-canonical), binds to the enzyme followed by the binding of the catalytic Mg_B_ ion and the closure of the active site loops. The initial reaction starts with the deprotonation of α-hydrogen by the ɛ-NH_2_ group of the lysine residue to form an intermediate carbanion. The resulting negative charge on the enol intermediate is stabilized by resonance with the carboxyl­ate oxygen and the magnesium ions. Following the formation of the keto-enol intermediate, the hydroxyl group on C3 is removed by the glutamate residue to form PEP. Once the reaction is complete, the catalytic metal ion (Mg_B_) is released first followed by the product [Figs. 1 ▸(*b*) and 1 ▸(*c*)]. The enolase–PEP complex is reported to favor the opening of the active site loops compared with the enolase–2PG-canonical complex in yeast, owing to the positive free energy change in the PEP-bound structure (Li & Hammes-Schiffer, 2019 ▸). It raises a pertinent question about reverse catalysis: how does 2PG, which strongly binds to the active site in the forward reaction, dissociate when formed as a product of PEP catalysis?

Our MtEno co-crystallization attempts with the substrate PEP yielded the 2PG-bound crystal. Two crystal structures of MtEno with the 2PG-bound form were elucidated. Of the two structures of MtEno-2PG, one harbored the widely reported 2PG-canonical pose whereas the other revealed 2PG in a novel binding pose [which we refer to as the alternate conformation (2PG-alternate)] in a manner that makes the otherwise unfavorable dissociation of the product from the active site thermodynamically feasible. Further investigation employing molecular dynamics (MD) and binding energy calculation studies of both conformations substantiated the notion that the 2PG product undergoes a conformational change to dissociate from the active site and facilitate the opening of the active site loops during the reverse reaction. We also describe the roles of each catalytic component in the reverse pathway, and how Mg_B_, a critical player of the forward catalysis, becomes dispensable in the reverse reaction.

## 2. Results

### 2.1. Structure of MtEno and comparison with conventional enolases

**Figure 2:**
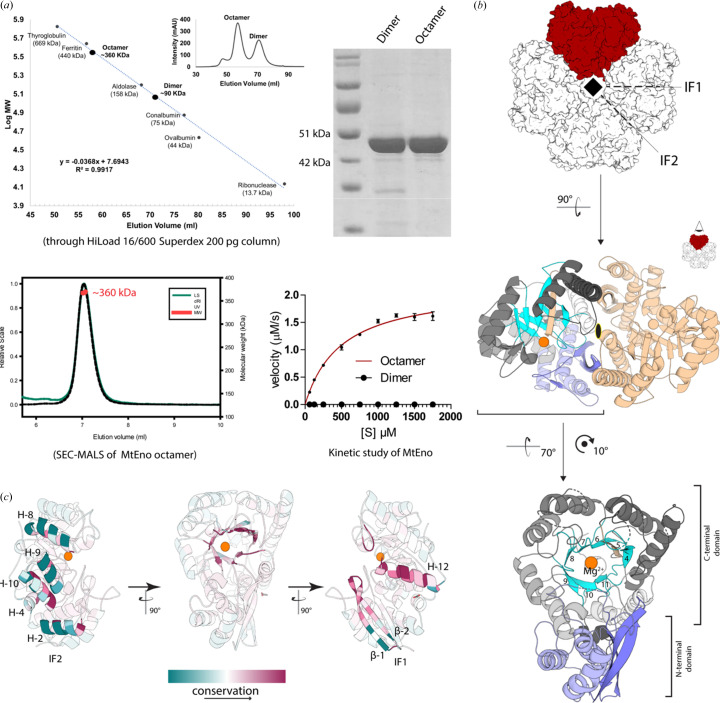
Structure and biochemical study of MtEno. (*a*) Biochemical study of MtEno. (Top) Size-exclusion chromatogram of MtEno indicates that the protein exists in both octameric and dimeric forms in solution. The octameric fraction was determined to be more pure than the dimeric fraction using SDS–PAGE. (Bottom-left) The homogeneity and size of the MtEno octameric fraction were further confirmed through SEC–MALS. (Bottom-right) Michalis–Menten kinetics with *K*_m_ = 495.7 ± 45.27 µ*M* for the reversible activity of octameric enolase against PEP, and with no measurable activity for dimeric enolase against PEP (*n* = 2 for technical duplicates). (*b*) Biological assembly of MtEno in the apo state (XRD structure shown). (Top) Conventional dimeric arrangement is highlighted in red and the IF1 and IF2 interfaces are marked with dashed lines; highlighted dimer is shown in cartoon (middle) with twofold rotational symmetry marked. (Bottom) Monomer architecture demarcated with N-terminal (light blue) and C-terminal (grayscale and cyan) domains. β-Strands near the catalytic core are labeled by their order in the polypeptide chain from N- to C-termini. β-5 is antiparallel to the rest of the barrel and hence is colored differently. (*c*) *Consurf* analysis of enolase monomer (Ashkenazy *et al.*, 2016 ▸). Structures involved in forming the IF2 (left), the C-terminal β-barrel (middle) or the IF1 (right) are shown with faded a structure of the rest of the monomer in the background. Red and teal indicate the most and least conserved, respectively.

*Mtb* H37Rv enolase was heterologously overexpressed with a 6×-His tag in the *Mycobacterium smegmatis* (*Msg*) mc^2^4517 strain and purified using a nickel–nitrilo­tri­acetic acid affinity column (Ni–NTA) and size-exclusion chromatography (SEC). The two distinct peaks in the SEC profile indicate that, although the majority of the protein exists in octameric form, a small fraction also exists as a dimer in solution [Fig. 2 ▸(*a*) (top)]. The absolute molecular weight and homogeneity of the octameric fraction was further confirmed through multi-angle light scattering (MALS) experiments [Fig. 2 ▸(*a*) (bottom left)]. The octameric state exhibited enzymatic activity by converting PEP to 2PG in the presence of 10 m*M* Mg^2+^ and 10 m*M* KCl with *K*_m_ = 497.5 ± 45.27 µ*M* [Fig. 2 ▸(*a*) (bottom right) and Table 1 ▸]. The dimer fractions showed no detectable activity. The octameric fraction was further used for structural studies. The structure of enolase in the apo state was determined using both cryoEM and X-ray crystallography (Tables 2 ▸, 3 ▸ and 4 ▸). The local resolution of cryoEM enolase structures ranges between 2.5 and 3.5 Å [Figs. S1(*a*)–S1(*c*)].

The crystal structure of MtEno apo protein shows a single protein molecule in the asymmetric unit and, on application of symmetry, an octameric assembly can be generated [Fig. 2 ▸(*b*)]. The subunits are arranged in a fourfold rotational symmetry, with two different types of interfaces between them [Fig. 2 ▸(*b*)]. One of them, described here as IF1, is similar to the interface observed in conventional dimers, whereas the other, IF2, exists in between two neighboring dimers and has only been reported in bacterial enolases (Wu *et al.*, 2015 ▸; Lu *et al.*, 2012 ▸; Ehinger *et al.*, 2004 ▸). MtEno has a topology similar to other enolase structures, a smaller N-terminal domain and a larger C-terminal domain. The catalytic site is circumscribed with a highly conserved discontinuous β-barrel made with parallel β strands β-4 and β6–β11 and a single antiparallel β-5 strand [Fig. 2 ▸(*b*) (bottom)]. While the tertiary structure of the monomers is nearly identical to those from the dimeric assemblies found in eukaryotes, there are subtle differences in the secondary structures of MtEno at the IF2 interface. The regions involved in the formation of the IF2 interface are not well conserved across all taxa, whereas those engaged in making IF1 are highly conserved [Figs. 2 ▸(*c*) and S2]. A phylogenetic analysis of enolase sequences reveals divergent evolution of the enzyme with higher eukaryotes such as humans and *Drosophila* at one end of the tree and *Mtb* at the other end [Fig. S3(*a*)]. Many of the interfacial residues of the IF2 interface within an octamer are conserved among the octameric enolases but not in the dimeric homologs (Fig. S2). Among the sequences analyzed, only prokaryotic species harbored enolases with an octameric assembly. Notably, all these species with octameric enolase are known pathogens that cause various diseases in humans, highlighting a significant variation in the enolase from *Mtb* and its natural host (humans) despite having a conserved active site (Wu *et al.*, 2015 ▸; Cork *et al.*, 2015 ▸; Lu *et al.*, 2012 ▸; Ehinger *et al.*, 2004 ▸).

The crystal and cryoEM structures of MtEno superimposed well with a low root mean square deviation (RMSD) of 0.58 Å for all Cα atoms [Fig. S3(*b*)]. Further, the MtEno crystal structure was superposed with its *Synechococcus elongates* counterpart with which it showed a low RMSD of 0.65 Å. The protein overall fold is highly conserved, except for a small conformational variation in loop 1 [Fig. S4(*a*)]. MtEno differs structurally from its eukaryotic counterparts by the presence of a slightly shortened helix H4, which contributes to the formation of IF2 (Fig. S2). We speculate that a shortened helix avoids steric clashes at IF2, enabling the MtEno dimers to tetramerize and ultimately exist as octamers. On the other hand, a longer H4 helix of eukaryotic enolases possibly causes steric hindrance, restricting the enzyme to a dimeric form. In comparison with human enolase-1, loop 3 of MtEno is shorter in length, a conserved feature of enolases in pathogenic bacteria [(Figs. S4(*b*) and S2].

The surface structure of the enolase plays an important role in binding the human plasminogen protein (Bergmann *et al.*, 2013 ▸; Xie *et al.*, 2023 ▸; Hemmadi *et al.*, 2022 ▸). The adaptive Poisson Boltzmann solver (APBS) surface potential of MtEno, unlike the human counterpart, is almost entirely negative except for Lys193, Lys194 and the lysine-rich C-terminal region [Fig. S4(*c*)]. Notably, human plasminogen interacts with the positively charged residues of MtEno, especially the lysine-rich C-terminal region (Rahi *et al.*, 2018 ▸, 2017 ▸).

### 2.2. Mapping the active site pocket

To elucidate the mechanism of MtEno activity, we set up co-crystallization experiments by incubating the protein with the substrate PEP. Although two crystal structures harboring different conformations of the product 2PG were obtained and elucidated (Tables 2 and 3), X-ray structures failed to provide PEP-bound MtEno. It prompted us to plunge-freeze MtEno–PEP solution and solve the structure using electron-cryomicroscopy (cryoEM) [Fig. S1(*b*); Table 4 ▸)].

**Figure 3:**
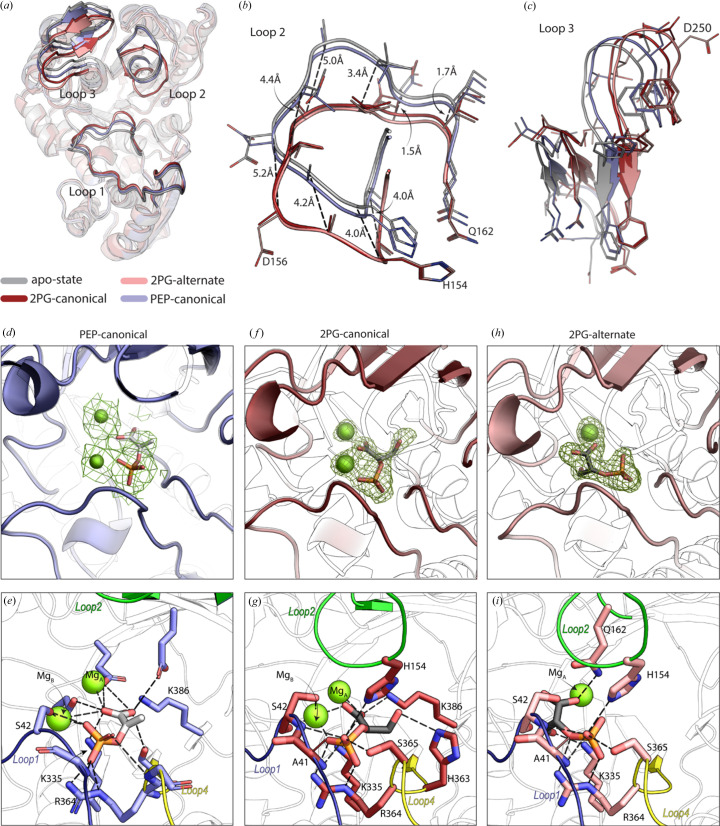
Structural comparison of the MtEno monomer at different stages of catalysis. (*a*) Cα superimposition of MtEno monomers solved in different catalytic stages: apo MtEno (gray), MtEno–2PG-canonical (brown), MtEno–2PG-alternate (pink) and MtEno–PEP (light blue) with loops 1, 2 and 3 highlighted. Focused sections of (*b*) loop 2 and (*c*) loop 3, with Cα displacement in MtEno–2PG-canonical when compared with the apo state, indicated by dashed lines. (*d*) CryoEM map curved around non-protein density at 7σ in which PEP and two Mg^2+^ ions fit in a similar orientation like that of 2PG-canonical and corresponding Mg^2+^ ions, shown in (*f*). (*e*) Hydrogen-bond-mediated interaction of PEP in canonical conformation within the binding pocket site. (*f*) |*Fo*| − |*Fc*| map showing electron density for 2PG-canonical at the 3σ level. (*g*) Hydrogen-bond-mediated interaction of 2PG-canonical. (*h*) |*F*_o_| − |*F*_c_| map showing the electron density for the 2PG-alternate at the 3σ level. (*i*) Hydrogen-bond-mediated interactions of the 2PG-alternate.

Superimposition of the PEP-bound cryoEM structure of MtEno and the crystal structure of its homolog from *Synechococcus elongatus* (PDB entry 5j04; González *et al.*, 2022 ▸) shows that the binding mode of PEP is conserved and it binds at the active site of the enzyme in the widely reported canonical conformation [PEP-canonical; Fig. S3(*c*)] located between the β-barrel core and loops 1 (Val37–Asp56), 2 (Gly151–Gln162) and 3 (Phe248–Thr262) [Fig. 3 ▸(*a*)].

Compared with the apo structure, 2PG/PEP-bound MtEno exhibits a global breathing motion in the C-terminal region towards the center of the domain and a displacement of loops 1–3 towards the catalytic site [Figs. 3 ▸(*a*)–3 ▸(*c*)]. This corroborates the reports of the apo-enzyme existing in an ‘open’ conformation and the holo-enzyme adopting a ‘closed’ conformation (Bergmann *et al.*, 2001 ▸; Avilán *et al.*, 2011 ▸). The motions of these loops are subtle in the PEP-bound state (Fig. S5), and although the overall structure resembles to that of the apo state of MtEno, the active site loops are partially closed in the presence of PEP. The two 2PG-bound structures depict closed active site loops and more pronounced deviations in loop 2, up to 5.2 Å as in the case of Asp156 [Figs. 3 ▸(*b*) and 3 ▸(*c*)].

Of the two structures of MtEno-2PG, one harbored the 2PG-canonical pose. MtEno–PEP-canonical [Figs. 3 ▸(*d*) and 3 ▸(*e*)] and MtEno–2PG-canonical [Figs. 3 ▸(*f*) and 3 ▸(*g*)] have similar orientations of the bound ligand. In both of these structures, Mg_A_^2+^ coordinates with the carboxyl groups of Asp241, Glu283 and Asp310; a water molecule; and the carb­oxy­lic group of 2PG/PEP. However, Mg_B_^2+^ coordinates weakly with the carb­oxy­lic and phosphate moieties of 2PG/PEP, the β-OH group of Ser42, and water molecules [Figs. 3 ▸(*e*) and 3 ▸(*g*)]. Hydrogen-bonding interactions with Arg364 in the highly conserved loop 4 further stabilize the associations via the phosphate moiety. Additionally, the carboxyl­ate group of 2PG/PEP interacts with both Mg^2+^ ions and residues Glu163, Lys386, Asp310 and Lys335 of the enzyme.

The other MtEno–2PG structure harbored 2PG-alternate [Fig. 3 ▸(*h*)]. Notably, 2PG in this alternate conformation has fewer hydrogen-bonding interactions in the active site due to the absence of catalytic Mg_B_^2+^. Mg_B_^2+^ plays an important role in the closing of active site loop 1 (Brewer *et al.*, 1998 ▸) and the neutralization of the negative charge of the phosphate group in 2PG-canonical [Fig. 3 ▸(*i*)]. Additionally, NZ of Lys386, which interacts with O2 of 2PG only in the 2PG-canonical pose does not form any interaction in alternate-2PG. We speculate that, by switching to this alternate conformation, 2PG facilitates its dissociation from the active site by weakening the loop 1 interaction (Ser42) with 2PG on its formation from PEP.

### 2.3. Tracking global motion in enolase through mutation at the active site and probable dispensability of Mg_B_^2+^ in the reverse reaction

**Figure 4:**
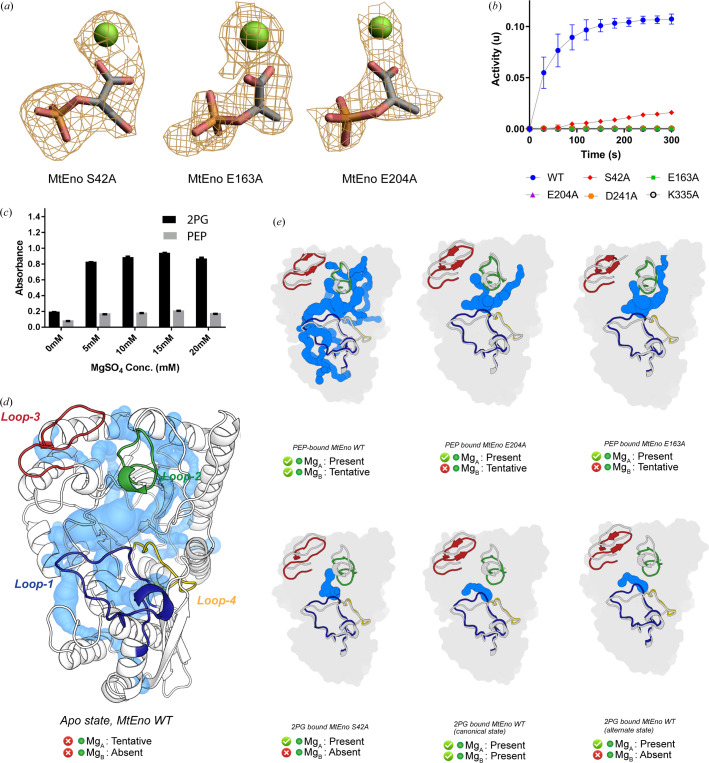
Water accessibility maps of MtEno at several stages of PEP to 2PG reaction catalysis. (*a*) |*F*_o_| − |*F*_c_| maps carved around ligand coordinates at the 3σ level. (*b*) PEP to 2PG conversion activity assay with MtEno WT and various point mutants. Error bars indicate the range for *n* = 2 for technical triplicates. (*c*) Activity assay of MtEno with varying metal ion concentration with 2PG/PEP as the substrate. (*d*) Cartoon representation of chain A of MtEno WT in the apo state with water-accessible tunnels shown: loops 1 (blue), 2 (green), 3 (red) and 4 (yellow) are highlighted. (*e*) Solvent-accessible tunnels shown for all structures solved. Loops 1–4 shown using the same representation as (*d*), structurally aligned to the corresponding loops in the MtEno WT Apo structure (gray loops). Ligands are hidden for clarity. Contours of the monomers are shown in the background. Whether the Mg^2+^ ions were modeled in the structures in (*c*) and (*d*) is indicated with a tick or cross along with the possibility of their occurrence in all states.

The forward reaction in enolase can be broken down into five interdependent yet sequentially distinct events: introduction of 2PG and Mg_B_^2+^ in the binding site, proton jump from the phosphate carbon to the Lys335 ɛNH_2_ group, keto-enol tautomerization, enolation reaction by Glu204, and exit of PEP and the water molecule from the active site [Fig. 1 ▸(*b*)]. Tautomerization resonance is accelerated on acidification of the carboxyl group of 2PG which is accomplished through its lone-pair-mediated coordination with both the Mg^2+^ ions and the transfer of a proton from His154 onto the nearest oxygen linked to the phosphate moiety. To find out if the exact reverse of this reaction occurs during the conversion of PEP to 2PG, we systematically mutated each of the residues Ser42, Asp241, Glu163, Glu204 and Lys335 to alanine, compared their activity with wild type MtEno, and elucidated the structures of three of them [Figs. 4 ▸(*a*) and 4 ▸(*b*)]. Notably, the K335A mutation disrupts the nucleophilic (proton) substitution, the E204A mutation defuncts enolation capacity, D241A should make the Mg_A_^2+^ coordination in the active site highly improbable and S42A should absolve the Mg_B_^2+^ coordination.

Interestingly, when PEP was soaked in the MtEno–S42A mutant crystals, the structure harbored the 2PG-canonical pose in the active site [Fig. 4 ▸(*a*)]. We analyzed the solvent accessibility in the protein structure based on tunnels and channels buried in the core of the protein by employing *CAVER* (Chovancova *et al.*, 2012*a* ▸). The water-accessibility channel tunnel of the MtEno S42A–2PG co-crystal structure was similar to that of MtEno Wt-2PG [Figs. 4 ▸(*d*) and 4 ▸(*e*)], corroborating our annotation of the substrate being 2PG. On the other hand, Mg_B_^2+^ was absent, possibly owing to the role of Ser42 in Mg_B_^2+^ coordination. However, conversion of PEP to 2PG even in its absence indicated that Mg_B_^2+^ may be non-obligatory for the reverse reaction. Moreover, the activity of MtEno–S42A is considerably reduced (∼5% of MtEno WT enzyme) but not completely abolished unlike other mutant enzymes which were non-functional [Fig. 4 ▸(*b*)]. Based on these results, it is plausible that, while Mg_B_^2+^ aids in the tautomerization, the presence of Mg_A_^2+^ makes its presence partially redundant. Further substantiating this, kinetic studies showed significant impact of increasing the metal ion concentration on the forward reaction but not on the reverse reaction [Fig. 4 ▸(*c*)].

We determined the structures of MtEno E163A and MtEno E204A and, as expected, both the proteins when co-crystallized with PEP had PEP in the active site and structurally resembled the PEP-bound form of MtEno WT [Figs. 4 ▸(*a*) and 4 ▸(*e*)]. Co-incidentally, no electron density peaks were observed for Mg_B_^2+^ in these structures [Fig. 4 ▸(*a*)]. This suggests that the introduction of PEP to the active site does not necessitate the presence of Mg_B_^2+^ in the active site, and although there are lesser interactions in the absence of Mg_B_^2+^, PEP aligns itself in the canonical conformation in the pocket as usual.

We also found that His154, Lys335 and Arg364 are the only residues involved in forming interactions with both 2PG and PEP in all conformations, and the interaction of His154 with the hydroxyl group of 2PG in the canonical conformation could drive the closing of loop 2 and subsequently loop 3 [Fig. 4 ▸(*e*)].

### 2.4. Proposed catalysis mechanism of PEP to 2PG

**Figure 5:**
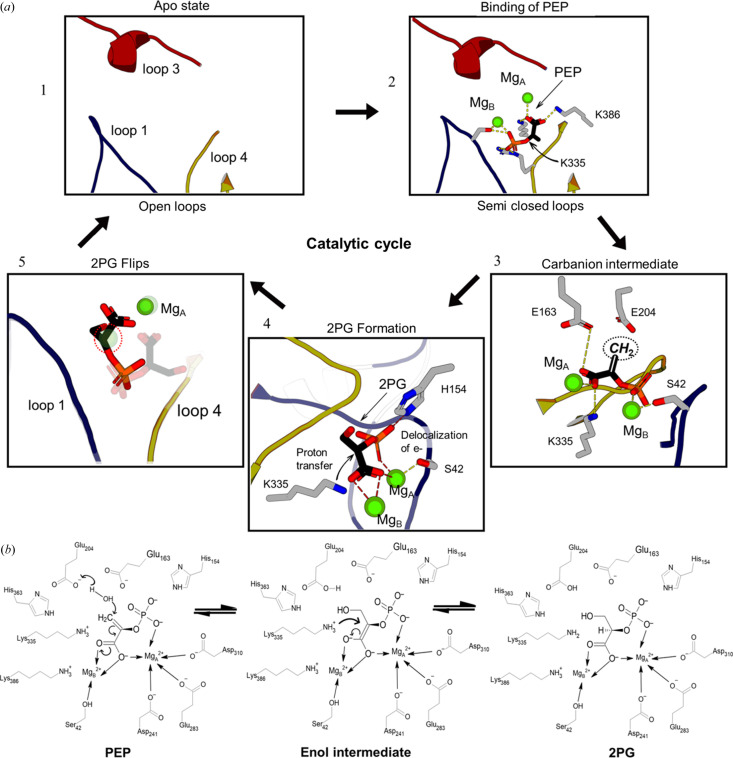
Mechanism of action and catalytic cycle for the reverse reaction. (*a*) Scheme of the proposed catalytic mechanism of the PEP to 2PG conversion. The reaction starts and completes one cycle in the apo state (top right). Hydrogen bonds are shown as yellow dashes. The position of Mg_B_ in the canonical 2PG state displaced in the alternate state is shown by a dotted circle. (*b*) Mechanism of reverse catalysis. Glu204 removes the proton from a water molecule to create a nucleophile (OH^−^) which attacks the *sp* ^2^ carbonyl group. The intermediate carbanion is stabilized by resonance. Following the formation of the carbanion intermediate, Lys335 donates a proton to C3 to form 2PG

Enolase follows a general acid–base catalysis in which the reaction depends on the protonation state of the catalytic residues of the active site (Liu *et al.*, 2000 ▸; Sims *et al.*, 2003 ▸). In order to understand the protonation state of the catalytic residues, we performed an empirical p*K*_a_ calculation through *PROPKA* which predicts the p*K*_a_ values of the ionizable groups from the 3D protein structure (Søndergaard *et al.*, 2011 ▸). Based on the solvent accessibility analyses, biochemical results and theoretical calculation of p*K*_a_ values, we propose the following steps in the enolase-mediated conversion of PEP to 2PG. The reaction starts with the introduction of PEP into the active site pocket which recalibrates the p*K*_a_ of residues around it [Figs. 5 ▸(*a*), S6(*a*)]. On binding of PEP, the slightly higher than usual p*K*_a_ values of Glu163 and Glu204 at ∼3.6 and ∼6.0 change to ∼5.7 and ∼8.1, respectively; this propels them to catalyse the oxidation of the terminal carbon of PEP via a water molecule, forming a carbanion intermediate [Figs. 5 ▸(*a*) and S6(*a*)]. The appearance of a hydroxyl moiety helps in forming hydrogen-bonding interactions with His154, bringing the loops inward. This causes a major shift in the bulk solvent exposure of many residues, altering their acidity further. Notably, the p*K*_a_ of Lys335 is estimated to be ∼11.5 in the MtEno WT apo state, unable to facilitate a reduction reaction on the carbanion intermediate. The p*K*_a_ decreases only slightly with the introduction of PEP to ∼10.5, but then drastically decreases with the formation of the carbanion derivative of 2PG, which can be roughly extrapolated from the p*K*_a_ in the 2PG-bound canonical form to ∼8.7 [Fig. S6(*a*)]. As a result, propensity for Lys335 to titrate its proton to the phosphate carbon increases. The formation of the carbanion is stabilized by the coordination of the 2PG carb­oxy­lic moiety with the Mg^2+^ ions. In the forward reaction, His154 has been reported to protonate the uncoordinated oxygen of the phosphate moiety and decrease the p*K*_a_ of the α hydrogen (Vinarov & Nowak, 1999 ▸). However, in an MtEno–2PG-canonical structure, the p*K*_a_ of the His154 sidechain is ∼8.2 and the p*K*_a_ of the average uncoordinated phosphate oxygen of the ligand is around ∼1.5, implying His154 may act as a nucleophile in the MtEno–2PG-canonical state, contributing more towards the reverse reaction than it does to the forward reaction [Figs. S6(*a*) and 5 ▸(*a*)]. Oxidation of Lys335 would subsequently reduce its electropositive contribution ability to the electrostatic field around the negatively charged 2PG. Finally, a highly negative environment created by the residues Asp241, Glu204 and Asp310 with their p*K*_a_ values ranging around 1.5, 0.3 and −2.3, respectively, results in a repulsive environment for the carb­oxy­lic group of 2PG, possibly causing it to flip [Figs. 5 ▸(*b*) and S6(*a*)]. This was further supported through the *in silico* alanine scanning mutagenesis of the active site residues, using the ABS-Scan server (Anand *et al.*, 2014 ▸) of all the conformations we solved in this study and this provided us with the quantity ΔΔG, which is conveniently close to the difference between the Gibbs free energy of the ligand-bound state and the apo state in the same conformation. The ΔΔG from the electrostatic interaction component is highly positive for the states with 2PG bound [Fig. S6(*b*]. The resultant electrostatic repulsion towards 2PG with an overview of the van der Waals radii clashes with 2PG against surrounding residues in the alternate conformation; we suggest that these clashes lead to the release of 2PG from the active site, preparing MtEno for yet another round of catalytic cycle [Figs. 5 ▸(*b*) and S6(*b*)].

### 2.5. Probing the role of the 2PG alternate conformation in reverse catalysis

**Figure 6:**
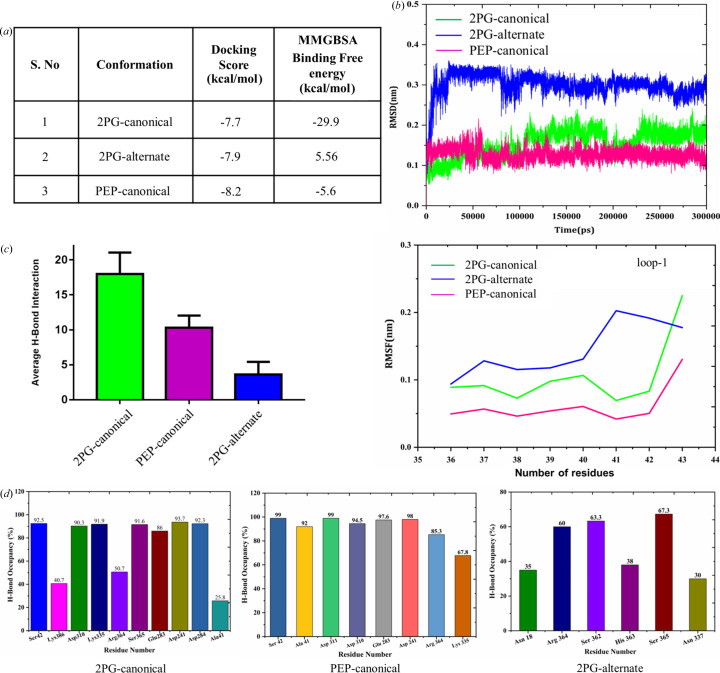
MD study of MtEno complexes. (*a*) Binding free energy values of all three complexes calculated through MM/GBSA. (*b*) The comparative RMSD and RMSF analyses of the loop 1 region in 2PG-canonical-bound (green), 2PG-alternate-bound (blue) and PEP-canonical-bound (magenta) MtEno complexes. (*c*) Average hydrogen-bonding interactions in all three complexes during dynamics. (*d*) Hydrogen-bond occupancy of catalytic residues in all three complexes.

As mentioned earlier, we elucidated two crystal structures of MtEno harboring different conformations of the product 2PG, the 2PG-alternate being exclusive to the reverse catalysis. This prompted us to investigate the role of the 2PG-alternate in the reverse catalysis. We calculated binding free energies of all the three complexes through MM/GBSA (generalized-born/surface area) (Genheden & Ryde, 2015 ▸; Ahinko *et al.*, 2019 ▸). The calculated binding free energies of the MtEno complexes exhibit contrasting results despite having similar docking scores. MtEno–PEP-canonical and MtEno–2PG-canonical possess negative binding free energy values of −5.6 and −29.9 kcal mol^−1^, respectively. However, the MtEno–2PG-alternate exhibits a positive binding free energy of 5.5 kcal mol^−1^, making it thermodynamically unfavorable for the 2PG-alternate to remain bound to the active site. We speculate that enolase catalyzes the conversion of PEP-canonical to 2PG-canonical which has an increased affinity towards the active site. However, on switching 2PG-canonical to 2PG-alternate, this affinity is greatly reduced [Fig. 6 ▸(*a*)], resulting in the dissociation of 2PG and its release from the active site as soon as the active site loops open.

As the three complexes show a significant difference in their binding free energies, we performed MD studies to observe the differences of the complexes. In particular, we were interested in finding any possible role of alternate-2PG in the active site loop opening in reverse catalysis by analyzing the loop flexibility, hydrogen-bonding interactions and metal ion coordination state. Interestingly, the MtEno–PEP-canonical, MtEno–2PG-canonical and MtEno–2PG-alternate complexes exhibit significant structural changes during the time course simulation, particularly in the active site loop region [Fig. S7(*a*)]. Loop 1 shows higher deviation in the MtEno–2PG-alternate than in the MtEno–PEP-canonical and MtEno–2PG-canonical complexes, indicating that 2PG-alternate loses its interactions with the residues of the loop 1 region and therefore enhances the loop flexibility to adapt the open conformation [Fig. 6 ▸(*b*)]. The higher fluctuation in the loop 1 region is largely due to the absence of Mg_B_ in the active site, and we hypothesize that the change in 2PG from the canonical to the alternate conformation deforms the Mg_B_ coordination, facilitating its release from the active site pocket. In MtEno–PEP-canonical, a relatively low number of hydrogen-bonding interactions were observed in the active site residues owing to the absence of the hydroxyl group in the PEP molecule compared with 2PG, hence we observed the loop 2 region in the open conformation [Fig. S7(*b*)], as also reported in yeast enolase (Li & Hammes-Schiffer, 2019 ▸). In addition, the Mg_B_ ion was found to have dissociated from the PEP molecule in the initial dynamics since it has weak co-ordination with the molecule as mentioned earlier. Since loop 3 is not directly involved in the interactions with the 2PG or PEP molecule, all three complexes have loop 3 in the closed conformation and do not undergo much flexibility or conformational changes during the dynamics [Fig. S7(*b*)]. Moreover, the average number of hydrogen-bonding interactions and hydrogen-bond occupancy of the active site residues are higher in MtEno–2PG-canonical than MtEno–2PG-alternate and MtEno–PEP-canonical throughout the dynamics [Figs. 6 ▸(*c*) and 6 ▸(*d*)]. We speculate that, owing to the decrease in metal ion coordination and hydrogen-bonding interactions compared with MtEno–2PG-canonical, loop opening is more favorable in MtEno–2PG-alternate, and during conversion of PEP to 2PG, the latter undergoes a conformational change to break the highly packed hydrogen-bonding interaction to facilitate release from the active site. As mentioned earlier, this switch of 2PG-canonical to 2PG-alternate is probably caused by repulsion of the carb­oxy­lic group of 2PG by the nearby residues. Once 2PG-alternate is formed, it mediates dissociation and release from the active site.

## 3. Discussion

*Mtb* utilizes fatty acid as a carbon source during an infection to survive in a nutrient-deficient environment (Segal & Bloch, 1957 ▸). The carbon flux produced during β-oxidation of fatty acids can be shifted towards gluconeogenesis, which generates glucose 6-phosphate and fructose 6-phosphate, the precursors for nucleotide and cell wall syntheses. Hence, gluconeogenesis is an essential pathway for *Mtb* to convert fatty acids into biomass and is likely to play a vital role in TB pathogenesis (Ganapathy *et al.*, 2015 ▸). Enolase, an essential protein of the glycolytic and gluconeogenic pathways, is also categorized as a moonlighting protein due to its diverse role in different cell types (Didiasova *et al.*, 2019 ▸). Mice immunization with MtEno has been reported to confer protection against *Mtb* infection (Rahi *et al.*, 2017 ▸), with an efficacy comparable to that of BCG (Horwitz *et al.*, 1995 ▸) and Ag85B (Karbalaei Zadeh Babaki *et al.*, 2017 ▸), presenting MtEno as a potential TB vaccine candidate.

In this study, our analysis pertaining to the evolution of MtEno along with the bacterial and eukaryotic enolases suggests that MtEno belongs to the group of octameric enolases, a feature exclusive to the pathogens. The crystal and the cryoEM structures (enzyme in solution, frozen) along with the biochemical studies clearly demonstrate that the functional unit of MtEno is an octamer. Bacterial enolases have evolved to adopt an octameric state, probably because catalytic loops in the dimeric enolases are unstable (Wu *et al.*, 2015 ▸). Of note, the kinetic parameters of bacterial octameric enolases and eukaryotic dimeric enolases are comparable, suggesting that existence in octameric form may be attributed to the diverse role of the enzyme in these pathogens, other than the usual catalysis.

Despite being a well studied enzyme, the mechanism of reversible catalysis in enolases remains largely uncharacterized. In this study, we present some sequential structural snapshots of reverse catalysis: (i) apo MtEno with open active site loops, (ii) MtEno-PEP (substrate-bound) with a partially closed active site loop, (iii) and (iv) two product-bound structures of MtEno *viz.* MtEno–2PG-canonical with a closed active site loop and MtEno–2PG-alternate with a closed active site loop. Although the crystal structures of both the MtEno–2PG exhibited closed loops, 2PG-alternate had a significantly lower number of interactions at the active site. Furthermore, the MD studies and binding free energy calculations indicated favorable dissociation of the product in the case of 2PG-alternate and increased flexibility of loops hinting at a possible loop opening. Perhaps when the loops were flexible, 2PG was released and we obtained a 2PG-alternate-bound crystal only when the loops had not opened. Taken together, our study suggests that 2PG-canonical switches to 2PG-alternate in order to dissociate and exit from the active site during reverse catalysis. Additionally, we found that in reverse catalysis, the role of magnesium as the cofactor is dispensable in comparison with the forward catalysis. This can explain the rationale for the rate of the reverse reaction being slower than the forward reaction in enolase (Hannaert *et al.*, 2003 ▸), since the proton abstraction in the first step of the reaction depends on the interaction of the metal ion with the substrate in forward catalysis (Liu *et al.*, 2000 ▸). In comparison with human enolase, MtEno has a shorter loop 3, and has an additional IF2 region, present only in octameric enolases. The absence of IF2 in human enolase makes it interesting to explore this region as a drug target.

Through an integrated structural, *in silico* and biochemical approach, this study has broadened our current understanding of the functional and mechanistic aspects of MtEno catalysis. The structural snapshots of MtEno in different catalytic states may assist in designing anti-TB inhibitors through a structure-guided drug discovery approach.

## 4. Material and methods

### 4.1. Sequence alignment and phylogenetic analysis

The MtEno (Uniprot ID P9WNL1) sequence was used as a search template for related homologous sequences in a non-redundant database. To cover enolases of varying divergences, a few selected sequences were taken from every slab of MtEno homologs, each slab representing sequences with *x*% − (*x* + 10)% sequence identity with MtEno. The sequences were aligned using the *clustal-O* program with iterative HMM clustering (Sievers & Higgins, 2018 ▸). The alignment was further used for functional and phylogenetic analysis.

The evolutionary history was inferred using the Maximum-Likelihood method and JTT matrix-based model (Jones *et al.*, 1992 ▸). The bootstrap consensus tree inferred from 100 replicates is taken to represent the evolutionary history of the taxa analyzed (Felsenstein, 1985 ▸). Branches corresponding to partitions reproduced in less than 50% bootstrap replicates are collapsed. Initial tree(s) for the heuristic search were obtained automatically by applying Neighbor–Join and BioNJ algorithms to a matrix of pairwise distances estimated using the JTT model, and then selecting the topology with a superior log likelihood value. This analysis involved 43 amino acid sequences. There was a total of 527 positions in the final dataset. Evolutionary analyses were conducted in *MEGA X* (Kumar *et al.*, 2018 ▸).

### 4.2. Construction, design and protein purification

The MtEno is coded by the ORF Rv1023 of *M. tuberculosis* H37Rv. The forward and reverse primers were constructed with gene-specific sequences along with codons for an N-terminal hexa-histidine tag (Table 5 ▸). The amplicon was directionally inserted into the entry vector pENTR following the manufacturer’s protocol (Invitrogen). Chemically competent *E. coli* DH5α cells were transformed with the entry clone carrying the recombinant ORF and the plasmid was isolated. The entry clone as well as the *Msg* mc^2^4517 shuttle expression vector pYUB1062 were independently double digested with restriction enzymes NdeI and HindIII. The insert released from the entry clone was purified and ligated into the linearized pYUB1062 to generate the recombinant plasmid pYUB-Rv1023. The successful integration of the insert into the expression vector and its directionality were confirmed by restriction digestion and Sanger sequencing. Site-directed mutagenesis was carried out using an Agilent QuikChange II XL site-directed mutagenesis (SDM) kit following the manufacturer’s protocol.

Pairs of primers used for different mutations are given in Table 5 ▸. The amplicons were treated with Dpn1 and then transformed into chemically competent *E. coli* DH5α competent cells. The SDM clones were confirmed by Sanger sequencing.

The recombinant construct containing the expression sequence (an N-terminal me­thio­nine and hexa-His tag followed by the Rv1023-specific amino acids) was electroporated into competent *Msg* mc^2^4517 cells for overexpression (Ahangar *et al.*, 2011 ▸). A single colony carrying the recombinant expression construct was revived in 10 ml of Luria Bertani (LB) broth supplemented with 0.05% Tween 80, 0.2% glycerol, and the antibiotics kanamycin (25 µg ml^−1^) and hygromycin B (100 µg ml^−1^). The volume was scaled up in primary and further in secondary cultures. All the cultures were grown at 310 K, 180 rpm for 24 h. The secondary culture (1.5 l) was induced with 0.2% acetamide when A600 reached 0.7. The cells were harvested at 10 000*g* for 20 min, 24 h post induction. The cell pellet was resuspended into the appropriate volume of buffer (20 m*M* Tris, pH 7.0, 150 m*M* NaCl, 5% glycerol, 25 m*M* imidazole and 10 m*M* MgSO_4_) along with one Complete Mini, EDTA-free protease inhibitor tablet (Roche Applied Science). Further, cell lysis was carried out using a cell disrupter (Constant Systems Ltd, UK) at 277 K and high pressure (25 000 psi). The lysate was centrifuged twice at 10 000*g* for 45 min at 277 K to remove unbroken cells and inclusion bodies. The supernatant obtained was loaded on a pre-equilibrated Ni–NTA. The column was then washed sequentially with resuspension buffer, 2 *M* NaCl, 50 m*M* imidazole in the same buffer to remove non-specifically bound impurities and finally the recombinant protein was eluted by increasing the concentration of imidazole to 300 m*M*. The eluted protein was concentrated to 5 mg ml^−1^ and further purified by SEC using a HiLoad 16/600 Superdex 200 prep grade column (GE Healthcare) in 20 m*M* Tris buffer pH 7 containing 150 m*M* NaCl, 25 m*M* imidazole and 1 m*M* MgSO_4_. The relevant protein sample was collected using the chromatogram and checked on 12% SDS–PAGE for the purity of the desired protein. Mass spectrometric analysis of the purified protein confirmed the identity of the protein as *Mtb* enolase. The same protocol was also followed for overexpression and purification of the mutant proteins.

### 4.3. Biochemical assays

The activity of MtEno was determined by monitoring the decrease in absorbance of PEP at 240 nm by following a method described elsewhere (Rahi *et al.*, 2017 ▸). Briefly, the reactions were carried out at 298 K in 200 µl of 0.1 *M* HEPES-10 m*M* KCl buffer pH 7.5 containing 10 m*M* MgSO_4_. The reaction was initiated by the addition of PEP (0.1–2 m*M*) and the decrease in PEP absorbance was monitored over a period of 45 s using a spectrophotometer (TCC-240A, Shimadzu Corp.). The level of decrease in PEP was calculated using a molar extinction coefficient of 1400 *M* ^−1^ cm^−1^. The data were plotted and the Michaelis–Menten kinetic parameters were calculated in *Prism* (version 6.0). The activities of the mutants were compared with wild-type enolase protein by measuring the decrease in PEP absorbance for 5 min.

For measuring the activity of MtEno with varying metal ion concentrations, Mg^2+^ was kept in the range 5–20 m*M* in the same buffer composition with 1 m*M* concentration of PEP and 2PG, respectively.

### 4.4. Crystallization and structure determination

The enolase, wild-type and mutant versions, obtained after SEC, were used for crystallization. The pre-crystallization test determined 9.5–10 mg ml^−1^ to be the optimum concentration of the enzyme for crystallization. The crystallization reservoir solution consisted of 25% PEG 3350, 0.2 *M* sodium acetate, 0.1 *M* Bis Tris pH 7 and the additive was 11.8 m*M* TCEP hydro­chloride. The protein crystallized within one week at 289 K in a hanging drop vapor-diffusion setup. The drop was set up in 24-well plates with a drop size of 3.4 µl (2 µl of protein, 1 µl of crystallization reservoir solution and 0.5 µl of additive). Finally, soaking the crystals in 50% MPD (in reservoir solution) for 5 min prior to mounting improved the diffraction quality of the crystals. Crystals of the enzyme–2PG complex were grown in a co-crystallization experiment under the same conditions that yielded apo enolase crystals except that the protein solution contained 0.5 m*M* 2PEP. The mixture was incubated for 10 min followed by manual plate set up. The mutant crystals in complex with 2PG/PEP were obtained by soaking the crystals in PEP for 2 min. For X-ray data collection, the crystals were mounted on Cryo-Loops (Hampton Research), rinsed in cryoprotectant solution (50% MPD in reservoir solution) and flash-cooled directly in a nitro­gen stream at 100 K. The datasets were indexed, integrated and scaled using *HKL*-2000 (Otwinowski & Minor, 1997 ▸). Data collection and statistics for all the structures are summarized in Table 2 ▸. The structure was solved by the molecular replace­ment method using the software *Phaser* (McCoy *et al.*, 2007 ▸) and the structure of *Synechococcus elongatus* (PDB entry 5j04) that shares 62% sequence identity with MtEno. A search model was generated from this template using the *Chainsaw* program (Winn *et al.*, 2011 ▸); the non-conserved residues were pruned to the last common atom of the MtEno specific amino acid sequence. The model was subjected to rigid-body refinement followed by 100 cycles of restrained positional refinement using a maximum likelihood target function. At this stage, model building was carried out based on electron-density maps (2|*F*_o_| − |*F*_c_|) at 1σ and (|*F*_o_| − |*F*_c_|) at 3σ (contour levels) using the program *Coot* (Emsley & Cowtan, 2004 ▸). Further, restrained refinement was performed using the structure refinement program *REFMAC* (Murshudov *et al.*, 2011 ▸). Some parts of the structure showed significant structural differences from the search model; therefore, extensive model building for these parts was performed to ensure the electron density maps corroborate with the model. Bulk solvent correction and anisotropic *B* factor scaling were incorporated in the refinement. Water molecules, 2PG, acetate and glycerol were modeled based on the (2|*F*_o_| − |*F*_c_|) at 1σ and (|*F*_o_| − |*F*_c_|) at 3σ (contour levels) electron-density maps. The stereochemistry of every residue was checked using *PROCHECK* (Laskowski *et al.*, 1993 ▸). The refinement statistics for all the structures are presented in Table 3 ▸. The rotational symmetry was checked using the program *LABELIT* (Poon *et al.*, 2010 ▸) before submission to the Protein Data Bank. The secondary structural elements were assigned using the program *DSSP* (Kabsch & Sander, 1983 ▸).

### 4.5. CryoEM data collection and structure determination

Freezing of apo–MtEno and PEP–MtEno at 3.5 mg ml^−1^ was accomplished with glow discharged Quantifoil holey carbon grids (R 0.6/1, Au 300 mesh) and a Vitrobot Mark IV set at 100% humidity and 16°C, with blotting for 3.5 s. Both the apo and the PEP–enolase data were collected in the Titan Krios at the National CryoEM facility, Bangalore, with a Falcon 3 detector in counting mode at 1.07 Å pixel^−1^ sampling with images exposed for 60 s, with a total accumulated dose of ∼27.70 e^−^ Å^−2^ and dose fractionated into 25 frames, with each frame having a dose of ∼1.1 e^−^ Å^−2^.

The datasets were processed in *RELION*-3.0 including its own full frame alignment (Scheres, 2012 ▸; Zivanov *et al.*, 2018 ▸). The summed images were subsequently used for automated particle picking with *Gautomatch* (Kai Zhang, MRC LMB, Cambridge), with a template derived from manual picking of the particles in *RELION*-3.0 and CTF was estimated with *Gctf* (Zhang, 2016 ▸). Particles were extracted with a box size of 320 pixels and subjected to two rounds of 2D classification followed by 3D auto-refinement, per particle CTF refinement, *B*-factor weighting with Bayesian polishing and refinement, and subsequent 3D classification in *RELION*-3.0 with *D*4 symmetry imposed. The nominal resolutions of apo–MtEno and PEP–MtEno were 3.1 and 3.2 Å respectively. Difference maps was calculated with a final unsharpened map to verify the presence of PEP in the active site. The local resolution of the maps was estimated with *ResMap* (Kucukelbir *et al.*, 2014 ▸). The 2PG-bound canonical state of the MtEno structure was used as the initial model and was docked in the *B*-factor sharpened map (*i.e.* after postprocessing) using *Chimera* (Goddard *et al.*, 2007 ▸) and further model building was performed with *Coot* (Emsley & Cowtan, 2004 ▸). The refinement of the model against the map was performed in real space with *Phenix* (Adams *et al.*, 2010 ▸). The presence of non-protein density in the active site of all eight monomers led us to model phospho­enolpyruvate (PEP) and Mg^2+^ in that location. The data collection, processing and refinement statistics are presented in Table 4 ▸.

## 5. Computational analysis

### 5.1. Preparation and docking studies of MtEno with 2PG/PEP

The crystal structures of enolase 2PG/PEP conformations were prepared using the standard protocol implemented in the *Schrodinger* software suite (https://www.schrodinger.com/). The optimized potential for the liquid simulations (OPLS)-2015 force field was used for energy minimization. This builds the missing residues in the structures to fetch the completeness of a protein for further studies. Simultaneously, the 2PG molecule was prepared using the *LigPrep* module and the structures were optimized by adding appropriate hydrogen bonds, and removing the steric clashes, ionization states and stereoisomers. The prepared structures of the MtEno and 2PG/PEP molecules were further docked by rigid-body docking without modifying the orientation or conformation of the 2PG/PEP using the *Glide* module (Ahmad *et al.*, 2020 ▸).

### 5.2. MMGBSA studies of MtEno 2PG-/PEP-bound complexes

The molecular mechanics energies combined with the Poisson–Boltzmann or generalized Born and surface area continuum solvation (MM/PBSA and MM/GBSA) are efficient methods to calculate the binding affinity of the canonical and alternate conformations of the 2PG/PEP molecule on MtEno protein. MM/GBSA was calculated for docked complexes using the *Prime* module implemented in the *Schrodinger* software suite. The MMGBSA binding free energy (Δ*G*_bind_) is calculated by the following equation,

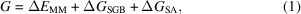

where Δ*E*_MM_ is the difference in the minimized molecular gas-phase interaction energies of protein–ligand complexes, Δ*G*_SGB_ denotes the surface calculation using the GB model of the complexes and Δ*G*_SA_ represents the solvent accessible surface area of the complexes.

### 5.3. Molecular dynamics simulation studies of MtEno 2PG-/PEP-bound complexes

The role of canonical and alternate conformations of the 2PG/PEP binding on MtEno protein was studied by MD simulation using the *GROMACS* (*Groningen machine for chemical simulations*) package (version 5.0; Van Der Spoel *et al.*, 2005 ▸). The co-crystallized canonical and alternate conformations of MtEno-complexed structures were considered for the MD studies for 300 ns time periods. The energy-minimized structure of the ligand molecule topology was generated using the *PRODrug* server (Schüttelkopf & Aalten, 2004 ▸) and was merged into the coordinates of enolase protein. The unit cell was defined in the shape of a cubic box and was filled with SPC216 water molecules. The distance between the solute and the box of the complex was set to 1.0 nm. To neutralize the solvated systems, Na ions were added and the systems were allowed to relax via energy minimization using the steepest-descent energy minimization algorithm until the maximum force under 1000 kcal^−1^ mol^−1^ nm^−1^. The complexes were equilibrated with two steps. The first step includes the 100 ps NVT ensemble (constant number of particles, volume and temperature) to stabilize the system at 310 K and the second step, the NPT (constant number of particles, pressure and temperature) equilibrium with 100 ps using a coupling reference pressure of 1 bar (atm). All bond lengths were constrained with the LINCS (linear constraint solver) algorithm (Hess *et al.*, 1997 ▸). The PME (particles mesh Ewald) electrostatic and periodic boundary conditions were applied in all directions (Kawata & Nagashima, 2001 ▸). The cut-off value of 9 Å for Coulomb interactions and 10 Å for van der Waals interactions were assigned. Pre-equilibrated systems of the complexes were subjected to MD studies for 300 ns time periods.

## 6. Structural analysis

All molecular visualization figures were prepared using *PyMOL* (version 2.5; Schrödinger) and its extensions *PROPKA* (version 3.4; Søndergaard *et al.*, 2011 ▸), *CAVER* (version 3.0; Chovancova *et al.*, 2012*b* ▸) and *APBS Electrostatics* (Jurrus *et al.*, 2018 ▸) (for surface electrostatic field representations). CryoEM density map [Fig. S1(*b*) of the supporting information] representations were made using UCSF *Chimera* (Pettersen *et al.*, 2004 ▸).

## 7. Data availability

The atomic coordinates and the structure factors have been deposited in the Protein Data Bank (https://www.rcsb.org) with the accession codes 7ckp, 7cll, 7clk, 7dlr, 7e4f and 6l7d. The cryoEM maps and the coordinates of the apo- and PEP-bound enolase have been deposited in the Protein Data Bank with the accession codes EMD-30988 and PDB-7e4x, and EMD-30989 and PDB-7e51, respectively.

## Supplementary Material

Supporting information file. DOI: 10.1107/S2052252523008485/lz5065sup1.pdf

PDB reference: *M. tuberculosis* enolase, 7ckp

PDB reference: *M. tubeculosis* enolase in complex with 2-phospho­glycerate, 7cll

PDB reference: *M. tuberculosis* enolase in complex with alternate 2-phospho­glycerate, 7clk

PDB reference: *M. tuberculosis* enolase mutant S42A, 6l7d

PDB reference: *M. tuberculosis* enolase mutant E163A, 7dlr

PDB reference: *M. tuberculosis* enolase mutant E204A complex with phospho­enolpyruvate, 7e4f

PDB reference: cryoEM structure of enolase from *M. tuberculosis*, 7e4x

PDB reference: cryoEM structure of PEP bound enolase from *M. tuberculosis*, 7e51

EMDB reference: enolase from *M. tuberculosis*, EMD-30988

EMDB reference: PEP bound enolase from *M. tuberculosis*, EMD-30989

## Figures and Tables

**Table 1:** Kinetic parameters of MtEno

*K*_m_ (µ*M*)	495.7 ± 45.27
*k*_cat_ (s^−1^)	12 ± 0.39
*k*_cat_/*K*_m_ (*M* ^−1^s^−1^)	24.21

**Table 2:** Data collection and processing statistics Values in the parentheses are for the highest resolution range.

	MPD bound	2PG canonical + 2PG	2PG alternate + 2PG	MtEno S42A + 2PG	MtEno E163A + PEP	MtEno E204A + PEP
Diffraction source	ESRF Beamline ID29	ESRF Beamline ID29	Rigaku FR-E+ SuperBright microfocus rotating copper anode	Rigaku FR-E+ SuperBright microfocus rotating copper anode	Rigaku FR-E+ SuperBright microfocus rotating copper anode	Rigaku FR-E+ SuperBright microfocus rotating copper anode
Wavelength (Å)	0.9775	0.9775	1.54178	1.54178	1.54178	1.54178
Space group	*I*422	*C*2221	*I*422	*I*422	*I*422	*I*422
Unit-cell parameters						
*a*, *b*, *c* (Å)	141.1, 141.1, 95.5	97.7, 197.3, 200.6	139.8, 139.8, 89.65	139.32, 139.32, 90.05	140.19, 140.19, 90.41	139.78, 139.78, 90.85
α, β, γ (°)	90, 90, 90	90, 90, 90	90, 90, 90	90, 90, 90	90, 90, 90	90, 90, 90
Matthew’s coefficient (Å^3^Da^−1^)	2.59	2.63	2.38	2.38	2.32	2.32
Subunits per asymmetric unit	1	4	1	1	1	1
Solvent content (%)	52.5	53.2	48.4	48.3	47.0	47.0
Temperature (K)	100	100	100	100	100	100
Detector	Dectris Pilatus3 6M	Dectris Pilatus3 6M	Rigaku R-AXIS IV^++^	Rigaku R-AXIS IV^++^	Rigaku R-AXIS IV^++^	Rigaku R-AXIS IV^++^
Diffraction limit (Å)	50.00–2.90 (2.95–2.90)	50.00–2.06 (2.02–1.99)	35.00–2.00 (2.07–2.00)	35.00–3.00 (3.11–3.00)	35.00–2.25 (2.29–2.25)	50–2.3 (2.34–2.3)
CC_1/2_	0.99 (0.72)	0.98 (0.60)	0.89 (0.58)	0.93 (0.72)	0.95 (0.78)	0.95 (0.73)
*R*_merge_ † (%)	7.0 (36.5)	14.4 (81.7)	8.0 (54.5)	12.7 (82.3)	7.1 (56.3)	11.2 (36.7)
Mean *I*/σ*I*(*I*)	23.6 (2.5)	10.1 (1.75)	13.3 (2.02)	15.8 (2.19)	20.3 (2.16)	22.2 (2.92)
Unique reflections	10414	121176	29616	8857	21190	20203
Multiplicity	21.6 (3.4)	9.1 (8.9)	4.7 (3.4)	8.4 (6.1)	5.0 (4.3)	12.7 (4.6)
Completeness (%)	94.7 (72.9)	91.8 (81.4)	97.9 (96.4)	97.1 (97.9)	98.3 (94.7)	99.4 (93.5)

† *R*_merge_(*I*) = ∑*_hkl_* ∑*_i_* |*I_i_* (*hkl*) − 〈*I*(*hkl*)〉|/∑*_hkl_* ∑*_i_I_i_* (*hkl*) for *n* independent reflections and *i* observations of a given reflection. 〈*I*(*hkl*)〉 is the average intensity of *i* observations.

**Table 3:** Refinement statistics

	MPD bound	2PG-canonical	2PG-alternate		MtEno S42A + 2PG	MtEno E163A + PEP	MtEno E204A + PEP
*R*_work_ (%)	24.2	17.1	15.9		19.9	16.3	17.6
*R*_free_ (%)	29.3	20.6	19.9		24.2	22.2	21.9
Average *B* factor (Å^2^)							
All atoms	74.7	29.8	28.9		59.3	33.4	31.6
Protein	74.8	29.1	28.0		59.3	32.9	31.2
Ligand	81.5	24.0	25.5		69.6	59.9	54.2
Metal ions	79.2	29.0	35.1		68.9	40.0	23.7
Water	54.3	36.4	38.1		43.1	36.7	31.2
Wilson *B* factor (Å^2^)	66.0	26.5	26.6		64.5	31.0	27.0
RMSD							
Bond angle (°)	1.4856	1.4984	1.4984		0.54	1.562	2.10
Bond length (Å)	0.0106	0.0115	0.0115		0.003	0.007	0.018
Ramachandran statistics (%)							
Allowed regions	91.8	96.2	97		95.4	96.1	97.1
Moderately allowed	7.7	3.4	3.0		4.2	3.5	2.6
Outliers	0.5	0.4	0		0.24	0.24	0.24
PDB entry	7ckp	7cll	7clk		6l7d	7dlr	7e4f

**Table 4:** Data collection, processing and refinement statistics for cryoEM

	Apo–enolase	Enolase–PEP complex
Data collection and processing		
Magnification (nominal)	75000×	75000×
Voltage (kV)	300	300
Electron exposure (e^−^ Å^−2^)	27.7	27.7
Nominal defocus range (µm)	−2.3 to −3.2	−2.3 to −3.2
Pixel size (Å)	1.07	1.07
Symmetry imposed	*D*4	*D*4
Initial particle images (No.)	153748	147184
Final particle images (No.)	81213	86231
Map resolution at FSC 0.143 (Å)	3.1	3.2
Map resolution range (Å)	2.5–3.5	2.5–3.5

Refinement		
Initial model used (PDB entry)	7cll	7cll
Map sharpening *B* factor (Å^2^)	−105.7	−120.7
Model composition		
Non-hydrogen atoms	24937	25033
Protein residues	3384	3384
Ligands	0	24
*B* factors (Å^2^)		
Protein	54.05	59.3
PEP		57.3
Mg^2+^		61.4
R.m.s. deviations		
Bond lengths (Å)	0.005	0.007
Bond angles (°)	0.564	0.689
Validation		
*MolProbity* score	1.92	1.92
Clashscore	9.45	9.39
Poor rotamers (%)	0	0
Ramachandran plot		
Favored (%)	93.6	93.5
Allowed (%)	6.1	6.2
Disallowed (%)	0.24	0.24

**Table 5:** Sequences of the primers used in the study

Rv1023_Fp^a^	5’-CACCCATATG *CACCATCATCATCATCAT*ATGCCGATTATCGAGCAGGTT-3’
Rv1023_Rp^a^	5’-TATAAGCTTCTATTTCGTCTCGCACGCGAACCGAGG-3’
Ser42Ala_Fp^a^	5’-CTCCCCGGTCGCGGCGCCCGACG-3’
Ser42Ala_Rp^a^	5’-CGTCGGGCGCCGCGACCGGGGAG-3’
Lys335Ala_Fp^a^	5’-TCCCGATCTGGTTCACCGCGACCAGCAACGCATTTG-3’
Lys335Ala_Rp^a^	5’-CAAATGCGTTGCTGGTCGCGGTGAACCAGATCGGGA-3’
Glu163Ala_Fp^a^	5’-GGCGCCACCATGAACGCTTGAATGTCGACAG-3’
Glu163Ala_Rp^a^	5’-CTGTCGACATTCAAGCGTTCATGGTGGCGCC-3’
Asp241Ala_Fp^a^	5’-GGCCGCCGCGGCCAGGGCCAG-3’
Asp241Ala_Rp^a^	5’-CTGGCCCTGGCCGCGGCGGCC-3
Glu204Ala_Fp^a^	5’-CGAAGCCGCCTGCGTCGCCCAGG-3’
Glu204Ala_Rp^a^	5’-CCTGGGCGACGCAGGCGGCTTCG-3
