# Supplementary material for: Structural snapshots of Mycobacterium tuberculosis enolase reveal dual mode of 2PG binding and its implication in enzyme catalysis
Source: IUCrJ. 2023 Oct 21;10(Pt 6):738–53. doi: 10.1107/S2052252523008485 (PMC10619443; doi:10.1107/S2052252523008485)
Supplement: Supplementary file 1 [file m-10-00738-sup1.pdf]

IUCrJ

Volume 11 (2024)

Supporting information for article:

Structural snapshots of *Mycobacterium tuberculosis* enolase reveal dual mode of 2PG binding and its implication in the enzyme catalysis

Mohammed Ahmad, Bhavya Jha, Sucharita Bose, Satish Tiwari, Abhisek Dwivedy, Deepshikha Kar, Ravikant Pal, Richard Mariadasse, Tanya Parish, Jeyaraman Jeyakanthan, Kutti R. Vinothkumar and Bichitra Kumar Biswal

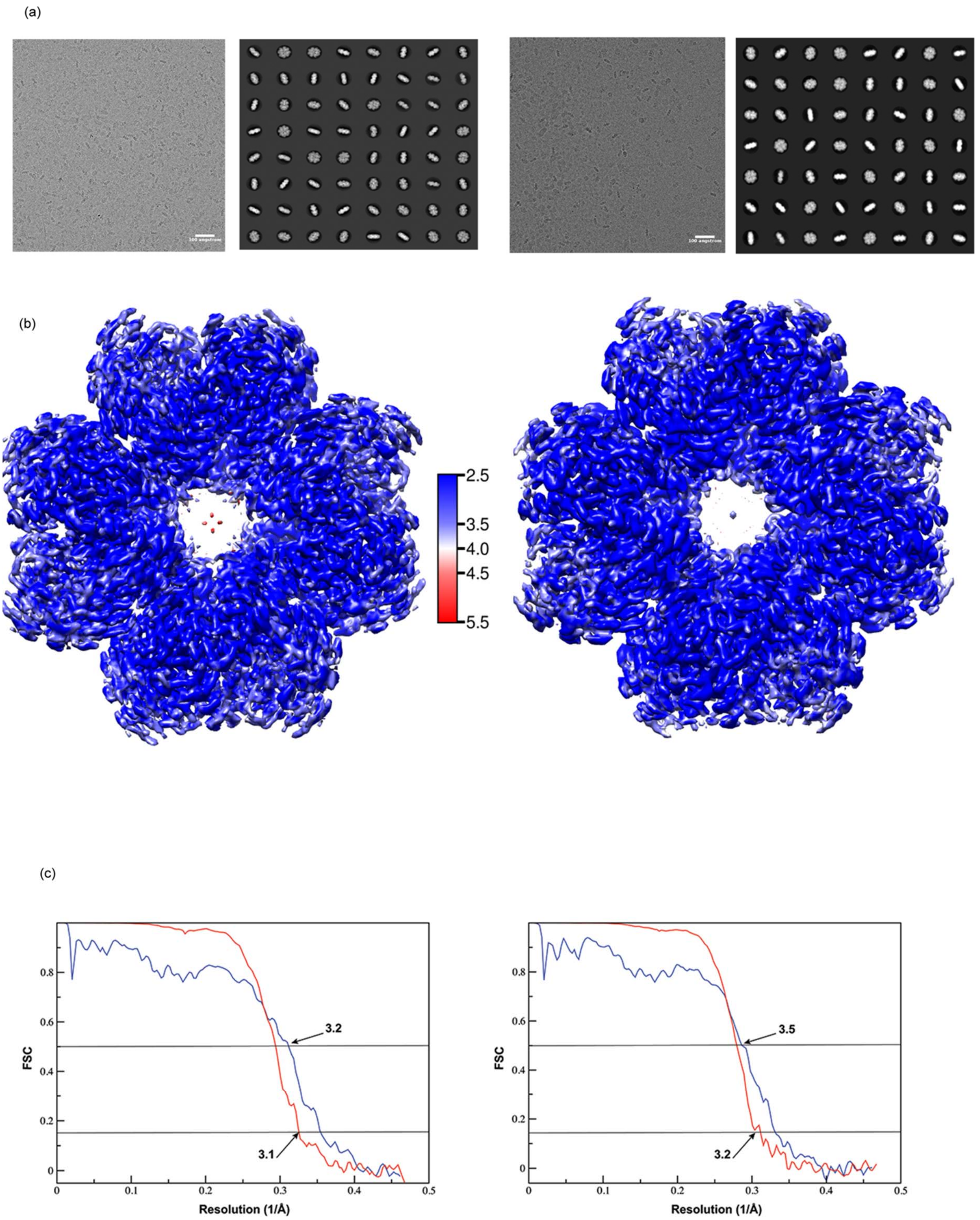

Supplementary Figure S1| CryoEM data and model summary for MtEno in Apo and PEP-bound states. a. Representative CryoEM Micrographs and corresponding 2D class averages of MtEno in Apo state *(left)* and in PEP-bound state *(right)*. Scale bar = 100 Å. **b.** Local resolution maps of MtEno in Apo *(left)* and PEP-bound *(right)* forms, with a majority of the structure resolved in the range of 2.5-3.0 Å. **c**. Respective fourier shell correlation (FSC) curves of two half-maps (red trace, evaluated at FSC of 0.143) and of the map and the model (blue trace, evaluated at FSC of 0.5) for apo *(left)* and PEP-bound *(right)* states.

IUCrJ (2024). 11, https://doi.org/10.1107/S2052252523008485 Supporting information, sup-1

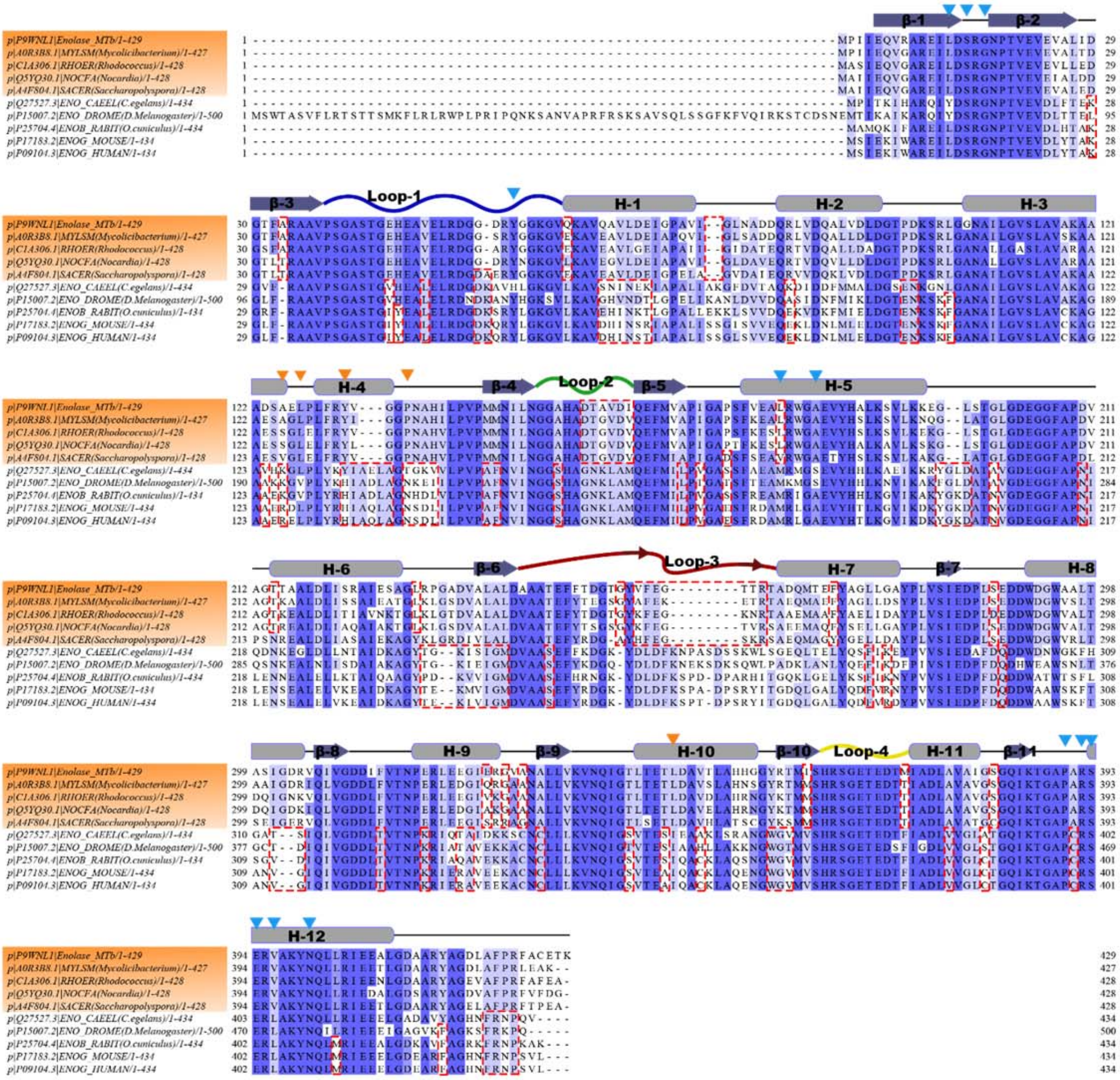

Supplementary Figure S2 - Multiple sequence alignment of Enolases from different organisms. Sequence titles highlighted in orange represent octameric enolases, while the rest are putatively dimeric in nature. Conserved differences between the two clades are highlighted with red dashed boxes. Sequences are shaded according to their degree of conservation, with darker shades representing higher conservation. Secondary structure assignments for MtEno are depicted above the alignment. Residues which are involved in forming the IF1 interface are pointed with blue triangles, and the ones involved in forming IF2 are pointed with orange triangles. Alignment was performed using clustalO algorithm with HMM clustering.

.

(a)

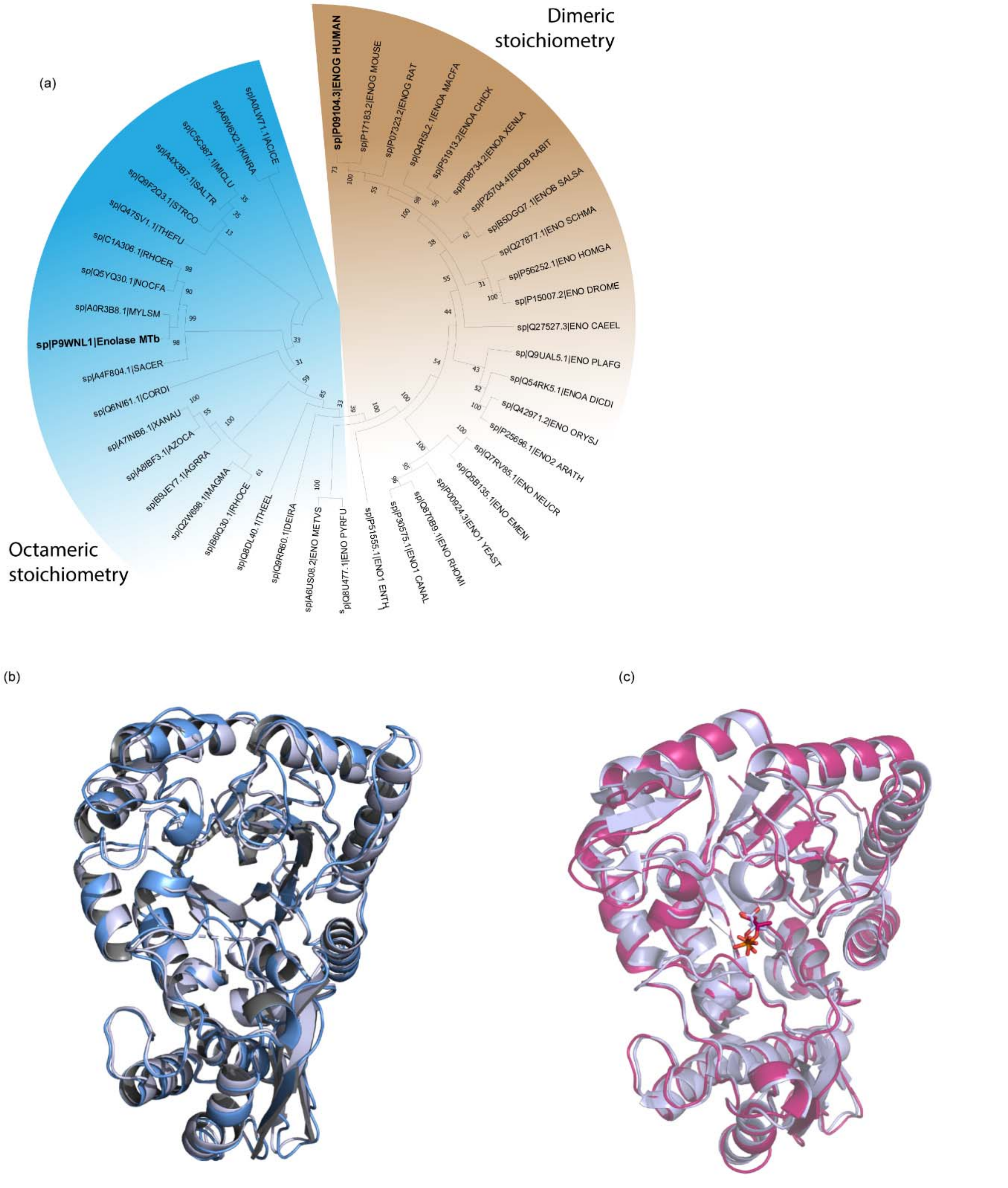

(b) (c)

Supplementary Figure S3| Phylogenetic and structural comparison of MtEno.

a. Radial phenogram of homologs of enolase across prokaryotes and eukaryotes. Homologs highlighted in a gradient from blue to brown have decreasing preferences to exist as an octameric functional state and increasing preference towards a dimeric state

b. Superimposition of cryoEM apo structure and crystal apo structure of MtEno. Crystal structure is depected in blue white while cryoEM is in teal color.

c. Superimposition of PEP bound MtEno cryoEM structure with *Synechococcus elongatus* PEP bound crystal structure. MtEno colored in pink while *Synechococcus elongatus* colored in blue-white.

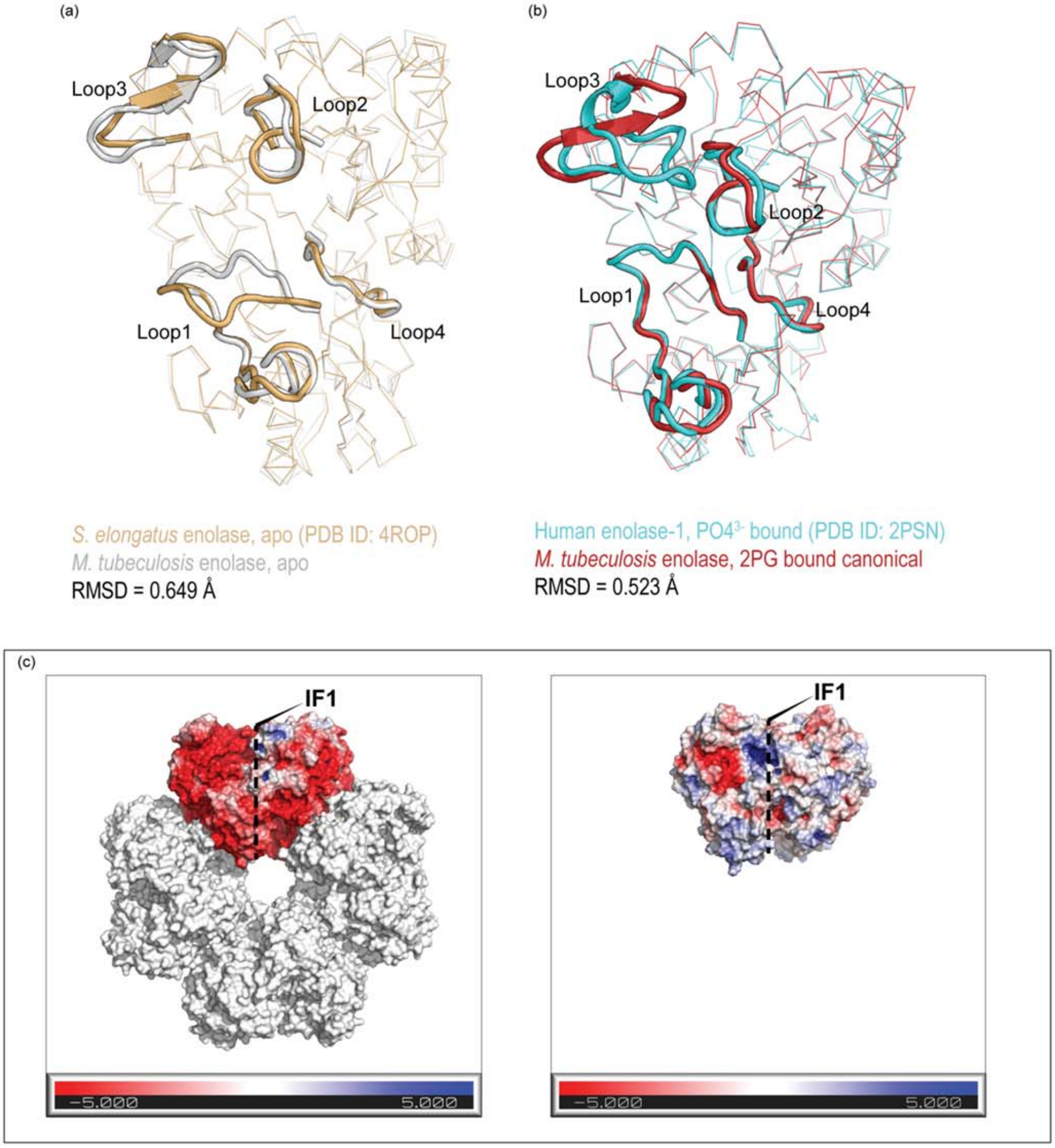

Supplementary Figure S4| Structural alignment of MtEno with dimeric and octameric enolases.
a. Structural superposition of *S. elongatus* enolase monomer (yellow, PDB ID: 4ROP) with MtEno monomer in apo state (white). RMSD = 0.649Å for 384 Cα atoms aligned.
b. Structural superposition of Human enolase-1 monomer (cyan, PDB ID: 2PSN) with MtEno monomer in the 2PG bound canonical state (red). RMSD = 0.523 Å for 331 Cα atoms aligned. Note that the loop-3 in human enolase is significantly elongated than the counterpart in MtEno.
c. APBS electrostatic surface of MtEno (apo state, left) and human enolase-1 (PDB ID: 2PSN, right), depicting a majority of negatively charged surface in the former and a mix of negatively and positively charged patches on the surface in the latter. The only positively charged patch on the surface of MtEno is found near the IF1 at the lysine rich regions.

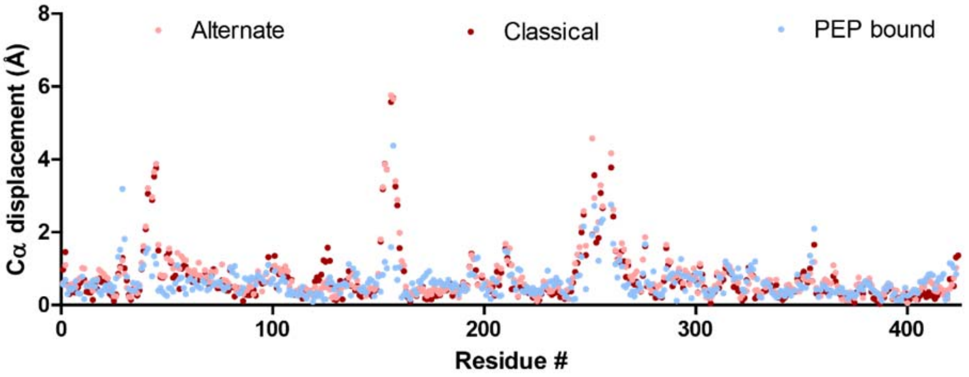

Supplementary Figure S5| Cα displacement profiles.

Cα positional comparison of chain A of MtEno WT solved in PEP bound state, canonical 2PG bound state, and alternate 2PG bound state with chain A of MtEno WT apo state. Chain A of each model was first structurally aligned to Chain A of apo state, and the transformed coordinates were used for displacement calculation.

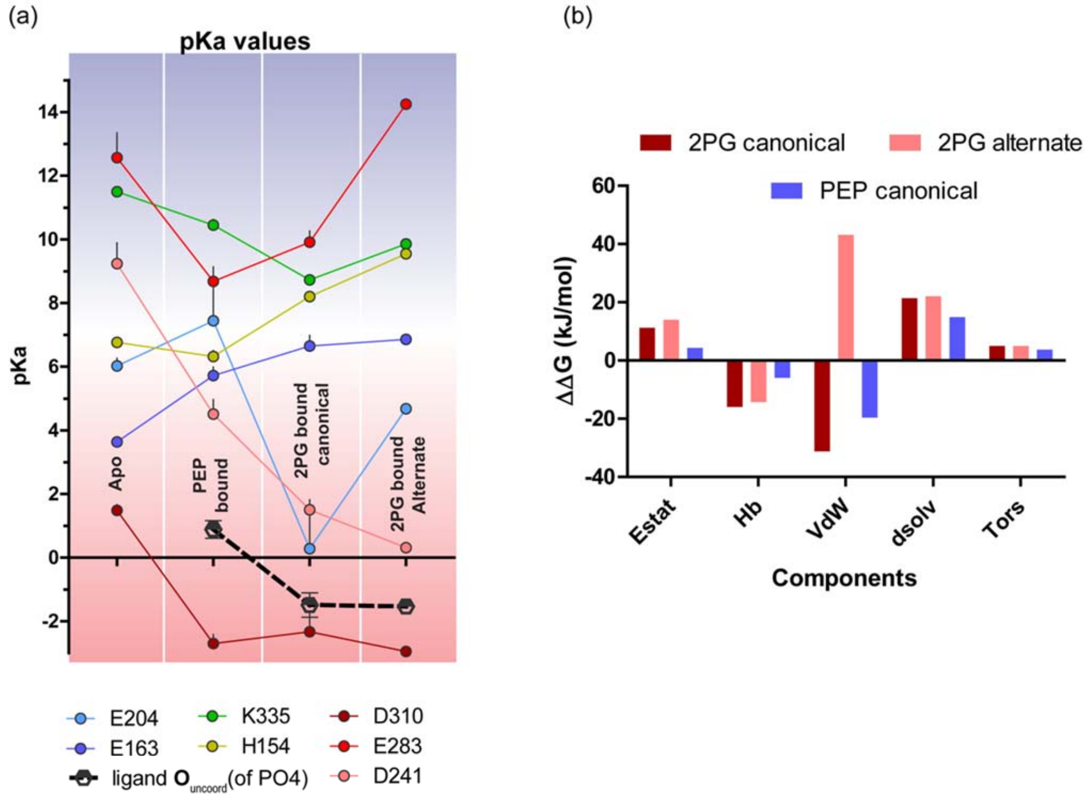

Supplementary Figure S6| pKa and ΔΔG during catalytic cycle.

a. pKa values of the residues involved in the catalytic conversion of PEP to 2PG. MtEno WT in apo and PEP bound models have 8 chains individually modeled in the density map, while 2PG bound canonical structure has 4 chains independently modeled, and error bars represent its variability in different chains. MtEno WT with 2PG bound alternate conformation structure has only one chain modeled in the asymmetric unit, and hence only one value of pKa was calculated.

b. Histogram for summation of ΔΔG values derived from *in-silico* alanine scanning mutagenesis of ligand binding in all three states in MtEno WT. Estat = Electrostatic interaction, Hb = Hydrogen bonds, VdW = Van der Waals forces, dsolv = desolvation, Tors = torsional strain.

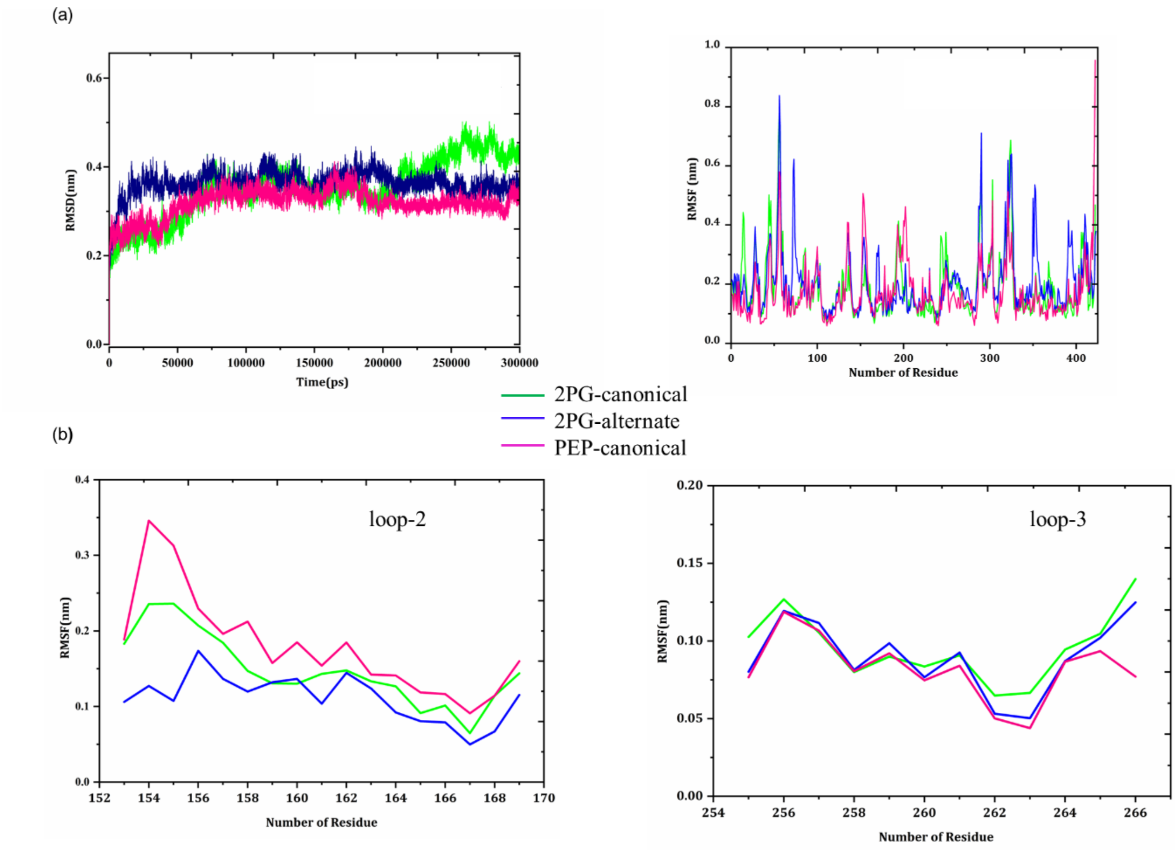

Supplementary Figure S7| Molecular dynamics study of MtEno complexes a. RMSD and RMSF analysis of canonical (Red), Alternative (Blue) conformation of 2PG and PEP (Magenta) MtEno complexes**. b.** The comparative RMSF analysis of loop-1 and loop-2 in classical (Red), alternative (Blue) 2PG and PEP (Magenta) MtEno complexes.
